# Spatial econometric analysis of health workforce distribution and its influencing factors in Inner Mongolia, China

**DOI:** 10.1371/journal.pone.0340381

**Published:** 2026-01-20

**Authors:** Jiajing Hu, Li Xu, Xuan Li, Sijia Liu

**Affiliations:** 1 School of Health Management, Inner Mongolia Medical University, Hohhot, China; 2 Inner Mongolia Mental Health Center (The Third Hospital of Inner Mongolia Autonomous Region, Brain Hospital of Inner Mongolia Autonomous Region), Hohhot, China; 3 Inner Mongolia Autonomous Region Center for Disease Control and Prevention (Inner Mongolia Autonomous Region Academy of Preventive Medicine), Hohhot, China; National Center for Chronic and Noncommunicable Disease Control and Prevention, Chinese Center for Disease Control and Prevention, CHINA

## Abstract

**Objective:**

Although health workforce equity has gained more attention, few studies have explored its spatial distribution and influencing factors in Inner Mongolia, a vast and diverse region of China. Existing researches often use simple geographic adjacency-based models that do not fully consider both location and economic factors. To address this, this study applies spatial econometric methods to examine the direct and indirect effects of influencing factors on health workforce distribution in Inner Mongolia from 2013 to 2022.

**Methods:**

Data were obtained from the Inner Mongolia Statistical Yearbook (2013–2022). Health workforce (HW) was measured by the number of health professionals per 1,000 persons. Spatial distribution and clustering patterns were analyzed using Global and Local Moran’s *I*. Four types of independent variables were selected: socioeconomic factors (per capita GDP and disposable income), demographic factors (population density and population growth), institutional environment (fiscal self-sufficiency rate), and supportive resources (beds density). A spatial panel econometric model was applied to assess both direct and indirect effects.

**Results:**

Significant spatial clustering of HW was found throughout the study period. High-high clusters were concentrated in the Hohhot-Baotou-Erdos region, while low-low clusters appeared in remote rural and pastoral counties. The Spatial Durbin Model (SDM) was chosen to explore the influencing factors. Direct effects showed that disposable income and bed density positively influenced HW within a county, whereas population density exhibited a significant negative impact. Indirect effects revealed that disposable income, fiscal self-sufficiency rate, and bed density also had positive spatial associations on HW in neighboring regions.

**Conclusion:**

Health workforce allocation in Inner Mongolia shows significant spatial disparities, with decreasing clustering over time, indicating reduced regional heterogeneity. Disposable income, bed density, and fiscal self-sufficiency positively affect HW and exhibit notable spatial associations, while population density has a negative impact. To optimize allocation, policies should enhance regional collaboration and resource sharing, increase fiscal support and promote medical alliances.

## Introduction

Health workers are an essential part of health systems and play a vital role in achieving universal health coverage (UHC) and primary health care (PHC). [1–3]. They are directly involved in providing medical services, promoting health, and improving population well-being. According to the World Health Organization (WHO), the health workforce includes a broad range of professionals such as doctors, nurses, midwives, pharmacists, and community health workers, as well as management and support staff who contribute to the functioning of healthcare systems [4,5]. In this study, we focus on those licensed professionals who deliver direct health services in institutional settings, excluding non-technical support staff, consistent with the classification adopted in the Inner Mongolia Statistical Yearbook [6].

Globally, the shortage and uneven distribution of health workers have become pressing challenges, particularly in low- and middle-income countries with limited health resources [3,7,8]. In China, disparities in health workforce are most evident between urban and rural areas, as well as among different regions [9–12]. These imbalances not only affect the quality and accessibility of healthcare services but also exacerbate social health inequalities [4,12,13].

The “Global Strategy on Human Resources for Health (GSHRH): Workforce 2030” highlights that effective management of health workforce is crucial to improving health outcomes and reducing inequalities in access to healthcare [1]. A key aspect of this management is the equitable distribution of health workers, which is vital for addressing gaps between urban and rural areas, as well as across different regions [3,14]. WHO advocates for policies that ensure health workers are allocated in a way that minimizes service gaps, especially in underserved and rural areas [1–3]. Such an approach is necessary for providing comprehensive healthcare coverage and promoting equity in healthcare provision across different populations.

The Chinese government has placed significant emphasis on optimizing the allocation of health workforce. The “Healthy China 2030” blueprint (2016) and the “14th Five-Year Plan for National Health Talent Development” (2022) both clearly outline the need to optimize health workforce distribution, with a particular focus on strengthening support for rural and grassroots healthcare personnel, aiming to improve the quality and level of healthcare services and achieve higher goals for public health [4,15].

Inner Mongolia, as a border region of China, faces unique challenges in the equitable distribution of health workforce [16]. Due to its vast territory and sparse population in many areas, the radius of healthcare service is often very large, complicating efforts to ensure that all residents have timely access to adequate healthcare [17]. Additionally, the uneven distribution of health workforce between urban and rural areas, as well as within the region, has widened the gap in accessibility and quality of healthcare. Urban centers typically concentrate more healthcare personnel, infrastructure, and advanced technologies, while rural and underdeveloped areas often suffer from insufficient services, fewer health workers, and limited facilities [18–21]. These imbalances lead to significant disparities in healthcare provision, leaving many rural residents without access to basic medical services and intensifying regional health inequalities [20,22]. Therefore, assessing the distribution and temporal changes of health workforce in Inner Mongolia, exploring the underlying driving factors and spatial effects, and investigating ways to optimize the allocation of health workforce is a key component in achieving universal health coverage.

Extensive researches have been conducted on the distribution and determinants of health workforce globally, employing various theoretical and empirical approaches. Previous studies have utilized tools such as the Lorenz curve, Gini coefficient, Theil index, and concentration index decomposition to assess equity in the distribution of health workforce across regions [4,5,10,11,23,24]. Spatial analysis methods, including spatial correlation analysis and spatio-temporal scan analysis, have been widely applied to explore geographic clustering and trends in health workforce allocation [12,25–29]. More advanced approaches such as regression model [30–32], spatial panel econometric models [33–36], and geographically weighted regression (GWR) [37–40] have been adopted to identify influencing factors and assess spatial heterogeneity. Building on these foundations, several studies have refined and expanded spatial approaches to health workforce research. Bai, et al. applied spatial panel econometric models to analyze workforce distribution in China, demonstrating the importance of capturing spatial dependence and heterogeneity to reveal inter-regional indirect effects [33]. Robin, et al. highlighted the value of GIS-based visualization in low-resource settings for identifying underserved areas, guiding local planning, and monitoring outcomes [26]. Zhu, et al. employed LISA statistics and geo-visualization tools to detect spatial clusters of workforce shortages, offering practical tools for identifying priority areas of intervention [40]. Asamani, et al. introduced a fiscal space framework for health workforce investment, emphasizing the integration of fiscal capacity analysis into workforce strategies to ensure sustainability and policy feasibility [41]. Together, these studies strengthen both the methodological and policy dimensions of spatial analysis and provide essential guidance for the analytical design of this research.

However, while previous studies have explored national-level patterns and employed various equity indices, relatively fewer have systematically examined the combined effects of socioeconomic, institutional, and infrastructural factors within a spatial econometric framework in regionally diverse Inner Mongolia. This study draws on two established frameworks from health economics and human resources research: (I) the analytical framework developed by Zurn, et al. [7] for understanding health workforce imbalances, and (II) the location decision model discussed by Dussault and Franceschini [13] which combines labor market dynamics with location theory to understand geographic differences in health workforce distribution. According to Zurn, et al. [7], health workforce imbalances are driven by a complex interplay of factors across six major dimensions: demand for and supply of health labor, health system characteristics, policies, resources (financial, physical, and human capital), and broad global factors (economic, geographic, sociodemographic, cultural). These dimensions influence workforce distribution through both direct and indirect mechanisms. Meanwhile, Dussault and Franceschini [13] emphasize that geographic imbalances result from health professionals’ location decisions, influenced by a utility-maximizing framework that accounts for individual, organizational, and environmental characteristics. These include not only financial incentives but also career development opportunities, community amenities, infrastructure, and personal or family considerations.

Based on these frameworks, our study classifies the influencing factors into four key categories that capture both structural and contextual determinants of health workforce distribution. Socioeconomic factors represent the level of economic development and income, which affect local living standards and the ability to attract health workers. Indicators such as GDP and income reflect regional economic capacity and are consistent with labor market theory [42], which suggests higher wages and better amenities attract skilled professionals. Demographic factors, including population density and population growth rate, reflect service demand and settlement patterns. Sparse or shrinking populations may struggle to support and retain health workers, while dense areas may face resource strain. The institutional factors represented by fiscal self-sufficiency, capture the financial autonomy of local governments to fund public health services. According to Asamani, et al. [41] fiscal capacity is a critical element of sustainable health workforce investment and policy feasibility. Furthermore, Benchimol, et al. [43] demonstrate that institutional characteristics and professional expertise can substantially affect how efficiently financial resources are translated into service outcomes, even when fiscal capacity is comparable across regions. Therefore, fiscal self-sufficiency serves as a proxy indicator of institutional capability in resource mobilization and utilization. Lastly, supportive resources, measured by beds density, reflect available health infrastructure, which influences both the efficiency of service delivery and professional working conditions. According to spatial equilibrium theory, these factors are expected not only to affect local health workforce directly but also to produce spillover effects in neighboring areas through mechanisms such as resource sharing, labor migration, or policy diffusion [44]. Therefore, this study applies spatial panel econometric models incorporating both direct and indirect effects.

Despite the wealth of existing research, studies specifically focusing on the spatial distribution of health workforce and its influencing factors in Inner Mongolia, China, remain limited. Most existing studies rely on geographic adjacency-based spatial weight matrices, which do not fully consider the combined effects of geographic location and economic development. However, Inner Mongolia is a large border region with diverse ethnic groups, unbalanced development, and challenges in healthcare access. These unique features highlight the need for a more suitable and region-specific spatial analysis. Therefore, by employing an economic-geographic nested spatial weight matrix and spatial panel econometric models, this study enhances existing literature by more accurately capturing regional heterogeneity and spatial dependence. The objective is to examine the spatio-temporal distribution of the health workforce in 103 county-level administrative units in Inner Mongolia from 2013 to 2022 and to explore the factors and inter-regional spatial associations influencing its allocation, providing scientific evidence for policymakers to optimize the allocation of health workforce and enhance the level of universal health coverage. Specifically, this study seeks to address the following research questions:

(1) What are the spatial distribution patterns of the health workforce in Inner Mongolia from 2013 to 2022?
(2) What are the key socioeconomic, demographic, institutional, and supportive factors influencing this distribution?
(3) Do these factors exhibit spatial interdependence across neighboring counties?

## Materials and methods

### Data sources and variable selection

This research utilized the panel data of 103 counties in Inner Mongolia from 2013 to 2022. All data were obtained from the Inner Mongolia Statistical Yearbook (2013–2022). According to the standards used in the Inner Mongolia Statistical Yearbook, health workers primarily include physicians, nurses, and health technicians, collectively referred to as health professionals [4,5]. Physicians include those holding a practicing physician certificate, encompassing both practicing physicians and assistants in China [5,10]. Nurses refer to registered nurses who have obtained a legally recognized nursing practice certificate [5,10]. Health technicians represent the workforce that assist medical staff in performing their duties within designated units or clinics to meet patient needs, including professionals such as pharmacists and radiologists [10]. In this study, the number of health professionals per 1,000 is used as the measurement indicator of health workforce [4,5,10].

Drawing on the frameworks of Zurn, et al. (7)and Dussault and Franceschini (13), and informed by spatial equilibrium theory [44], we classify the influencing factors into four key categories: socioeconomic, demographic, institutional environment, and supportive resources. This classification is grounded in both theoretical rationale and data availability (see Table 1 and S1 Table).

**Table 1:** Definitions and descriptive statistics of variables.

Variable type	Variable name	Measurement	Units	Abbr.	*N*_obs	Mean	Median(Q1,Q3)	SD	Min	Max
Dependent variables	Health workforce	Health professionals per 1,000 population	persons/1,000	HW	1030	8.11	5.98(3.96,9.33)	7.90	0.92	82.52
Independent variables	Socioeconomic Factors	GDP per capita	CNY/person	GDP	1030	102169.70	66078.88(36711.04,137779.70)	94501.88	16923.87	663200.10
		Disposable income per capita	CNY/person	Income	1030	26696.05	18095.00(34099.00,59551.00)	10886.22	9011.00	59551.00
	Demographic Factors	Population density	persons/km²	PD	1030	166.55	45.70(8.78, 120.25)	345.72	0.16	2635.22
		Population growth	per 10,000 population	PG	1030	−0.11	−0.03(−0.24,0.11)	3.68	−27.59	28.21
	Institutional Environment	Fiscal self-sufficiency ratio	ratio	Fiscal	1030	0.37	0.23(0.12,0.52)	0.32	0.04	1.91
	Supportive resources	Hospital beds per 1,000 population	beds/1,000	Bed	1030	5.94	4.65(3.30,6.85)	4.93	0.83	73.96

Socioeconomic Factors: GDP per capita and disposable income per capita were chosen as indicators.

Demographic Factors: These include population density and population growth.

Institutional Environment: The fiscal self-sufficiency ratio, calculated as general public budget revenue divided by general public budget expenditure, was selected to reflect regional financial capacity.

Supportive resources: The number of hospital beds per 1,000 population was used as an indicator.

### Research hypotheses

Drawing on labor market [42] and spatial equilibrium theories [44], as well as established health workforce frameworks [7,13], this study proposes testable hypotheses on the key factors influencing the distribution of health workforce across counties in Inner Mongolia.

#### H1. Socioeconomic development (GDP).

Regions with higher GDP per capita are expected to exhibit a greater density of health workforce (positive effect, +). According to labor market theory [42], economically developed regions can offer higher compensation, better infrastructure, and improved living conditions, which attract and retain skilled health professionals [7,45].

#### H2. Disposable income (Income).

Disposable income per capita is hypothesized to have a positive effect (+) on health workforce distribution. Following Benchimol and Qureshi [46], as income levels rise, both individuals and local governments tend to shift preferences from basic subsistence toward higher-quality and specialized healthcare services, increasing the demand for health workforce expansion.

#### H3. Population density (PD).

Population density is expected to have a positive association (+) with the health workforce. Denser populations imply greater healthcare demand and more efficient service delivery, making these regions more attractive for health professionals under spatial equilibrium conditions [13,44,45].

#### H4. Population growth (PG).

Population growth is hypothesized to have a positive effect (+) on the health workforce. Areas with increasing populations generate higher healthcare needs, which stimulate demand for additional health personnel. Conversely, declining or stagnant populations may discourage workforce allocation [13].

#### H5. Fiscal self-sufficiency (Fiscal).

Fiscal self-sufficiency is expected to exhibit a positive effect (+) on the health workforce. Consistent with Asamani, et al. [41] and Benchimol, et al. [43], greater fiscal capacity enhances the ability of local governments to finance healthcare services and sustain workforce investment, reflecting stronger institutional capacity and resource mobilization efficiency.

#### H6. Supportive resources (Bed).

The number of hospital beds per 1,000 population is hypothesized to have a positive relationship (+) with the health workforce. According to spatial equilibrium theory [44], regions with better healthcare infrastructure provide favorable working environments and higher operational efficiency, thereby attracting and retaining more health professionals [7,13].

In summary, all independent variables are expected to exhibit positive relationships with health workforce density, though the magnitude of effects may vary across regions due to spatial associations related to resource sharing, labor mobility, and policy diffusion.

### Methods

#### Spatial weight matrix.

In the field of spatial econometrics, Waldo Tobler’s First Law of Geography states that “everything is related to everything else, but near things are more related than distant things.” [47] Based on this principle, the primary task in spatial econometric analysis is to construct a spatial weight matrix to measure the spatial correlation among variables, ensuring that the model can capture the spatial interactions between regions. To better characterize the spatial distribution of health workforce and its influencing factors in Inner Mongolia, this study employed an economic-geographic nested matrix. This matrix not only considers the geographic proximity between regions but also incorporates economic factors, reflecting the relationship in health workforce allocation among regions with different economic levels. The specific form is as follows:


$$w_{ij}=\frac{\text{1}}{\left|e_{i}-e_{j}+\text{1}\right|}\times \text{exp}(-\beta d_{ij})$$

Where $e_{i}$and $e_{j}$ represent the economic levels (GDP per capita in CNY/person) of county *i* and *j*, respectively. $d_{ij}$denotes the geographical distance between county *i* and *j* (in kilometers), and *β* is the decay coefficient reflecting the diminishing influence of geographic distance on spatial interaction. To avoid division by zero when two counties have identical GDP, a small constant (numerical stabilizer) of 1 was added to the economic distance. To assess the robustness of the findings, several alternative spatial weight matrices that incorporate both geographic and economic dimensions were additionally constructed: (1) an inverse-distance matrix $w_{ij}^{\left(\text{1}\right)}=\frac{\text{1}}{d_{ij}}$; (2) an exponential-decay economic–geographic matrix

$w_{ij}^{\left(\text{2}\right)}=e_{i}\times e_{j}\times \text{exp}(-\beta d_{ij})$; (3) an economic-distance adjusted matrix$w_{ij}^{\left(\text{3}\right)}=\frac{e_{i}\times e_{j}}{d_{ij}}$; and (4) a nested economic–geographic matrix$W=W_{d}•diag\left(\frac{e_{\text{1}}}{e},\frac{e_{\text{2}}}{e},\cdots \frac{e_{n}}{e}\right)$, where $W_{d}$is the inverse-distance matrix$\;(w_{ij}^{\left(\text{1}\right)})$,$e=\sum_{j=\text{1}}^{n}\frac{e_{j}}{e}$. These alternatives allow us to test whether the results are sensitive to the inclusion of economic similarity in spatial interactions. Regarding the decay parameter *β*, while *β* = 1 often used as a conventional reference value in the absence of prior information [48], we also tested alternative values (*β* = 0.5, 2.0) to evaluate parameter sensitivity. The resulting spatial weight matrix was row-standardized.

#### Spatial autocorrelation analysis.

To analyze the spatial distribution characteristics and correlations of health workforce across 103 counties in Inner Mongolia, this study utilized both the Global Moran’s *I* and Local Moran’s *I* indices to conduct spatial correlation tests. These methods can reveal the patterns of aggregation or dispersion of health workforce and identify spatial clusters or anomalies in specific localities.

1. Global Moran’s *I*

The Global Moran’s *I* test assesses overall spatial autocorrelation, indicating whether there is a significant spatial clustering of health workforce across all counties [12,27,49]. The formula for Global Moran’s *I* is expressed as follows:


$$I=\frac{n\sum_{i=1}^{n}\sum_{j=1}^{n}w_{ij}(x_{i}-\overset{―}{x})(x_{j}-\overset{―}{x})}{\sum_{i=1}^{n}\sum_{j=1}^{n}w_{ij}\sum_{i=1}^{n}{\left(x_{i}-\overset{―}{x}\right)}^{2}}$$

Where $x_{\text{i}}$ and $x_{j}$ represent the number of health professionals per 1,000 population in counties *i* and *j*, respectively. $\overset{―}{x}$ denotes the average number of health workforce density across all counties. $w_{ij}$ is the spatial weight matrix and *n* is the total number of counties. The value of *I* ranges from −1–1. A value of *I* > 0 indicates positive spatial autocorrelation, suggesting clustering of similar values, while *I* < 0 suggests negative spatial autocorrelation, indicating dispersion. An *I* value approaching 0 implies a random distribution of spatial attributes. The magnitude of the Moran’s *I* value indicates the strength of the spatial correlation, thus, a higher absolute value of *I* represents a stronger correlation [49].

2. Local Moran’s *I*

The Local Moran’s *I* test examines local spatial clustering phenomena, highlighting spatial autocorrelation in individual county and their neighbors [12,27,29,49]. The formula for Local Moran’s *I* is given as follows:


$$I_{i}=\frac{(x_{i}-\overset{―}{x})\sum_{j}^{n}w_{ij}(x_{j}-\overset{―}{x})}{\sum_{i=1}^{n}{\left(x_{i}-\overset{―}{x}\right)}^{2}/n}$$

The results from the Local Moran’s *I* calculations can be visualized using Local Indicators of Spatial Association (LISA) cluster maps. Based on the Local Moran’s *I*, each county was classified into one of four types. High-high (HH) clusters refer to counties with high health workforce density surrounded by similarly high-density neighbors (hot spots). Low-low (LL) clusters refer to counties with low density adjacent to others with low density (cold spots). High-low (HL) and low-high (LH) clusters are considered spatial outliers, where a county’s value differs from its neighbors. The LISA maps used color coding to display these patterns, and only statistically significant clusters (*p* < 0.05) were shown. These maps helped identify regions of concentration or shortage in workforce allocation [29,49]. The spatial distribution and spatial correlation analysis were conducted using ArcMap 10.8(ESRI, Redlands, CA, USA). Local Moran’s I statistics were computed using 999 permutation tests, and significance was evaluated based on permutation *p*-values with (or without) multiple-testing adjustment.

#### Spatial panel econometric model.

Spatial panel econometric models integrate the principles of spatial econometrics and panel data analysis, providing a robust method for examining datasets with both temporal and spatial dimensions. This approach is particularly effective for analyzing interdependencies and dynamic changes among regions over time [35,36]. By employing spatial panel econometric models, evaluates the direct effects of independent variables on the dependent variable, as traditional regression models do, but also captures spatial indirect effects, revealing the interrelations between regions [33]. The models utilized in this research include the Spatial Lag Model (SLM), Spatial Error Model (SEM), and Spatial Durbin Model (SDM), with their specific formulations detailed below.


$$y_{it}$$

Where $y_{it}$ is the dependent variable, specifically *ln*HW (the logarithm of health workforce) for county *i* at time *t*, $x_{it}^{\prime }$ is a row vector of independent variable for county *i* a*t* time *t*. $w_{ij}$ is the element in the spatial weight matrix, ***β*** is the column vec*t*or of regression coefficients for the independent variable. *ρ* is the spatial autoregressive coefficient (|*ρ*| < 1), which reflects the effect of the spatially lagged dependent variable and ensures model stability and convergence. *θ* is the coefficient for the spatially lagged independent variables, reflecting the impact of neighboring regions’ independent variables on the dependent variable in the current county, and *λ* represents the spatial error autoregressive coefficient, which indicates spatial correlation in the error terms. $\mu_{i}$denotes the individual effect, $\gamma_{t}$ denotes time effect, $u_{it}$ and ${\;\epsilon }_{it}$ are the error terms.

According to the model specifications: When *ρ* ≠ 0, *θ* = 0, and *λ* = 0, the model is Spatial Lag Model (SLM), indicating spatial dependence among the dependent variables. When *ρ* = 0, *θ* = 0 and *λ* ≠ 0, the model is Spatial Error Model (SEM), suggesting spatial correlation in the error terms. When *ρ* ≠ 0, *θ* ≠ 0, and *λ* = 0, the model is Spatial Durbin Model (SDM), indicating that both the dependent and independent variables exhibit spatial dependence.

In spatial econometric models such as SDM, the estimated coefficients cannot be directly interpreted as marginal effects due to the presence of spatial lags. To address this, we followed the approach proposed by Parent and LeSage [50], which decomposes the total effects of each independent variable into direct and indirect components. The direct effect reflects the impact of a change in an independent variable within a county on its own dependent variable. The indirect effect captures the influence of that change on neighboring counties’ dependent variables through spatial interactions. The total effect is the sum of both direct and indirect effects. These effects were computed using partial derivative matrices based on the estimated spatial autoregressive coefficients and spatial weight matrix.

To address heteroscedasticity and enhance data stationarity, logarithmic transformations were applied to all variables except for population growth. The stationarity of the data was assessed using the Levin-Lin-Chu (LLC) unit root test. The results, as presented Table 3, confirmed that all variables were stationary. To control for the impact of multicollinearity among variables, two approaches were employed. First, variance inflation factors (VIF) and tolerances (1/VIF) were calculated based on ordinary least squares (OLS) regression results. Variables with VIF > 10 or 1/VIF < 0.1 were removed from the model. Second, correlation coefficients were examined, with values below 0.85 suggesting an acceptable level of correlation [33]. The results showed that all VIF values were below 10 and all correlation coefficients were less than 0.85, indicating that there was no multicollinearity issue in this study (Table 2 and Table 3). A *p* < 0.05 was considered statistically significant. All the analyses performed in STATA 14.1 software.

**Table 2:** Correlation analysis between independent variables.

	*ln*GDP	*ln*Income	*ln*PD	PG	*ln*Fiscal	*ln*Bed
*ln*GDP	1.0000					
*ln*Income	0.7076^***^	1.0000				
*ln*PD	−0.0898^***^	0.1105^***^	1.0000			
PG	−0.0521^*^	−0.0015	0.0075	1.0000		
*ln*Fiscal	0.7563^***^	0.5702^***^	0.3474^***^	0.0119	1.0000	
*ln*Bed	0.4710^***^	0.6333^***^	0.1662^***^	−0.1024	0.4458^***^	1.0000

***p ＜ 0.01, **p ＜ 0.05, *p ＜ 0.1

**Table 3:** Collinearity and unit root tests.

Variables	VIF	1/VIF	LLC	*p*-value
*ln*GDP	5.1	0.195943	−18.3218	0.000
*ln*Income	2.75	0.363275	−37.7603	0.000
*ln*PD	1.84	0.544264	−24.5666	0.000
PG	1.04	0.965321	−39.8299	0.000
*ln*Fiscal	4.05	0.246734	−14.1708	0.000
*ln*Bed	1.74	0.573334	−22.1043	0.000

## Results

### Descriptive analysis

**Fig 1:**
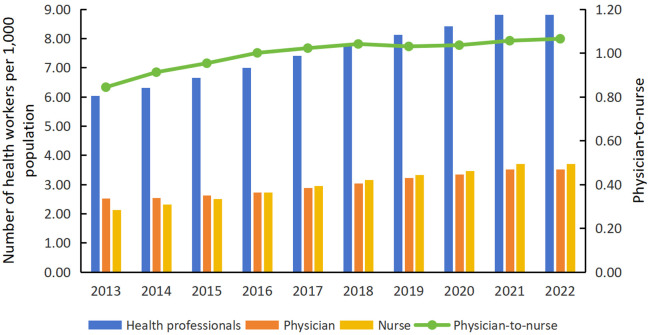
The changes in health workforce in Inner Mongolia from 2013 to 2022.

**Fig 2:**
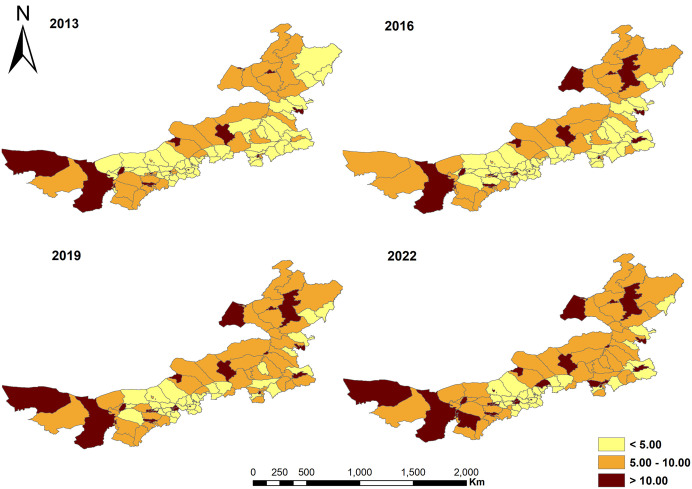
Spatial distribution of health workforce density in counties of Inner Mongolia, 2013-2022.

Fig 1 illustrated the changes in the health workforce in Inner Mongolia from 2013 to 2022. During this period, the number of health professionals, physicians, and nurses per 1,000 population showed an increasing trend, with nurses experiencing the highest growth rate at 73.76%, while doctors climbed by 38.85%. From 2013 to 2015, the physician-to-nurse ratio was inverted, reflecting a shortage of nurses. Starting from 2016, the numbers of nurses and physicians have reached near parity, with a physician-to-nurse ratio of 1:1.00. By 2022, this ratio had further improved to 1:1.06, indicating progress in addressing the nursing workforce gap. Regions with health professional higher densities, initially concentrated in the western and northeastern areas as well as certain administrative centers of cities (or leagues), have gradually expanded from both the eastern and western regions towards the central area. Meanwhile, regions with lower densities have steadily decreased, with such areas mainly concentrated in the central region by 2022 (Fig 2). To visualize the spatial distribution patterns of the independent variables, maps of *ln*Income, *ln*Bed, *ln*Fiscal, and *ln*PD were plotted (see S1 Fig).

To compare the temporal variation of the main independent variables, S2 Fig presents the time-series trends of *ln*Income, *ln*Bed, *ln*Fiscal, *ln*PD, and PG from 2013 to 2022. Overall, *ln*Income and *ln*Bed exhibited a steady upward trend, indicating continuous improvement in residents’ income and health resources. *ln*Fiscal showed moderate fluctuations over time, while *ln*PD and PG remained relatively stable with only minor year-to-year variations.

### Spatial autocorrelation analysis

From 2013 to 2020, the global Moran’s *I* values of HW were positive, with *z* values exceeding 1.96 (all *p* < 0.05). This indicates a significant spatial aggregation in the distribution of health workforce in Inner Mongolia. During this period, although the global Moran’s *I* fluctuated, it generally showed a downward trend, suggesting that the spatial aggregation of health workforce has overall decreased (Table 4).

**Table 4:** Global Moran’s *I* of HW in Inner Mongolia.

Year	Moran’s *I*	*z*-value	*p*-value
2013	0.446	7.384	0.000
2014	0.170	3.809	0.000
2015	0.233	4.431	0.000
2016	0.298	5.194	0.000
2017	0.087	1.992	0.046
2018	0.358	6.053	0.000
2019	0.369	6.112	0.000
2020	0.334	5.592	0.000
2021	0.321	5.353	0.000
2022	0.253	4.259	0.000

**Fig 3:**
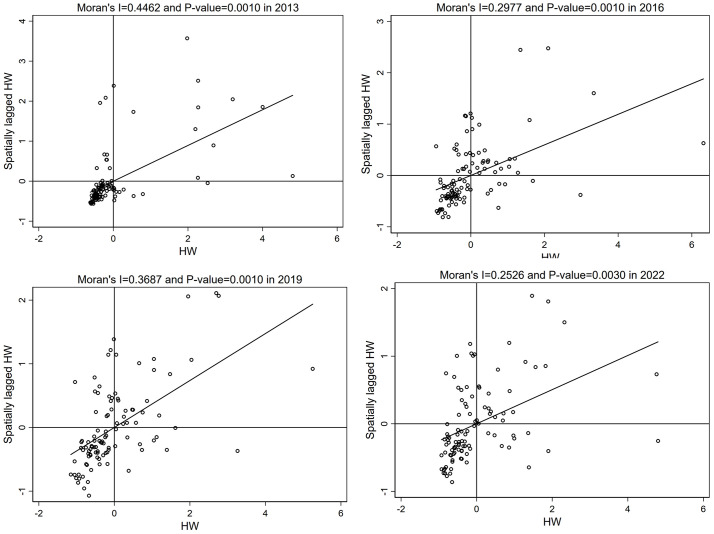
Moran’s *I* scatterplots for HW in 2013, 2016, 2019, and 2022 (In the Moran’s I plots, Quadrant I represents High–High (HH) clusters, Quadrant II Low–High (LH), Quadrant III Low–Low (LL), and Quadrant IV High–Low (HL)).

This study generated Moran’s *I* scatterplots to analyze the distribution of health workforce for the years 2013, 2016, 2019, and 2022 (Fig 3). The results revealed that areas characterized by high-high clusters (Quadrants I) and low-low clusters (Quadrants III) were predominant, indicating a significant spatial autocorrelation. In 2013, high-high and low-low clustering were notable. Over time, while positive spatial autocorrelation persisted, the clustering effect gradually weakened, showing a trend towards dispersion. This indicates that during the study period, the spatial distribution of health workforce shifted from a state of pronounced clustering to a more balanced distribution.

**Fig 4:**
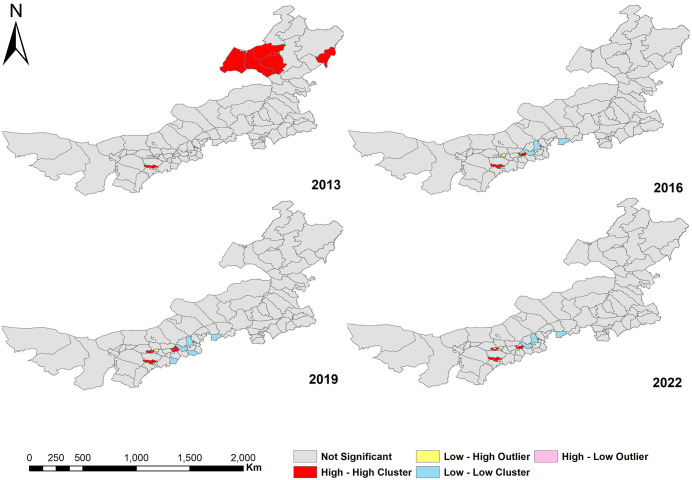
The LISA cluster map of HW in 2013, 2016, 2019, and 2022 (Areas with significant local spatial autocorrelation are color-coded: High–High (HH) clusters in red, Low–Low (LL) clusters in blue, Low–High (LH) outliers in yellow, High–Low (HL) outliers in pink, and non-significant areas in gray).

The LISA cluster map of health workforce distribution revealed distinct spatial patterns (Fig 4). In 2013, high-high clustering area was identified in the northeastern city of Hulunbuir. However, in subsequent years, high-high clusters have predominantly shifted to the central and western regions, including Yuquan District, Huimin District, and Saihan District in Hohhot City; Dongsheng District and Kangbashi District in Ordos City; and Qingshan District and Jiuyuan District in Baotou City. These areas, primarily located in the administrative centers of their respective cities (or leagues), are characterized by relatively developed economies and dense populations. Conversely, low-low clusters were largely situated in the central region, such as Taipusi Banner in Xilin Gol League and Zhuozi County and Chahar Right Back Banner in Ulanqab City. These areas are mainly rural or pastoral, with lower economic development levels. This highlights the long-standing inadequacy in the allocation of health workforce in these regions (Table 5).

**Table 5:** Local spatial autocorrelation analysis of HW in 2013, 2016, 2019, and 2022.

Year	Spatial correlation	Cities	Counties	Num
2013	H-H	Ordos, Hulunbuir	Dongsheng District, Hailar District, Jalainur District, Morin Dawa Daur Autonomous Banner, Ewenk Autonomous Banner, Chen Barag Banner, Xin Barag Left Banner, Xin Barag Right Banner	8
2016	H-H	Hohhot, Ordos	Huimin District, Yuchuan District, Saihan District, Dongsheng District, Kangbashi District	5
	L-L	Xilingol, Ulanqab	Zhuozi County, Chahar Right Back Banner, Taibus Banner	3
2019	H-H	Hohhot, Baotou, Ordos	Xincheng District, Huimin District, Yuchuan District, Saihan District, Kundulun District, Qingshan District, Jiuyuan District, Dongsheng District, Kangbashi District	9
	L-L	Ulanqab, Xilingol, Hohhot	Qingshuihe County, Zhuozi County, Chahar Right Back Banner, Fengzhen County, Taibus Banner	5
2022	H-H	Hohhot, Baotou, Ordos	Huimin District, Yuchuan District, Saihan District, Qingshan District, Jiuyuan District, Dongsheng District, Kangbashi District	7
	L-L	Xilingol, Ulanqab	Zhuozi County, Chahar Right Back Banner, Taibus Banner	3

### Spatial panel data analysis

#### Selection of spatial econometric models.

Based on the above analysis, the distribution of health workforce in Inner Mongolia exhibited a significant spatial correlation. To better measure the influencing factors and spatial effects, spatial econometric models were constructed. Initially, Lagrange Multiplier (LM) tests and their robust versions were conducted. According to the criteria proposed by Anselin and Florax [51], these tests were used to determine the most suitable model among SLM, SEM, and SDM. The results showed that all four LM tests were significant, indicating both spatial lag effects and spatial error autocorrelation exist within the sample data (Table 6). Given that the SDM can simultaneously capture both effects, it was preliminarily identified as the appropriate model for this study.

**Table 6:** LM test and Robust LM test.

Test	LM-value	*p*-value
LM-Lag	141.025	0.000
Robust LM-Lag	4.325	0.038
LM-Error	221.01	0.000
Robus LM-Error	84.31	0.000

Then, Hausman test was performed to determine the type of model effects. The results showed the Hausman test was significant ($\chi^{2}$(13) = 38.54, *p* < 0.001), rejecting the null hypothesis of the random effect model and indicating that the fixed effect model is more suitable. Further evaluation using log-likelihood values and likelihood ratio (LR) statistics revealed that both spatial fixed effects (LR = 46.17, *p* < 0.001) and time fixed effects (LR = 753.21, *p* < 0.001) were significant, supporting the selection of a two-way fixed effect model that incorporates both spatial and temporal dimensions.

Finally, LR and Wald tests were employed to verify whether the SDM could be simplified to the SLM or SEM by testing the joint significance of the spatially lagged independent variables. As shown in Table 7, the LR tests for both SLM (*p* = 0.0083) and SEM (*p* = 0.0134) were statistically significant. The Wald test for SLM was also significant (*p* = 0.0333), while the result for SEM (*p* = 0.0819) was marginal. These results confirmed that the SDM should not be simplified to either an SLM or SEM. Furthermore, regression models were constructed for the SAR, SEM and SDM. The results demonstrated that the SDM has the lowest *σ*^*2*^ and the highest goodness-of-fit *R*²among the models (Table 8). Thus, in terms of model performance, the SDM was identified as the most optimal choice. In summary, this study selected the SDM with a two-way fixed effect to explore the factors influencing the distributional differences in health workforce.

**Table 7:** LR test and Wald test.

Test	Statistics	*p*-value
LR-Lag	17.29	0.0083
LR-Error	16.07	0.0134
Wald-Lag	13.69	0.0333
Wald-Error	11.22	0.0819

**Table 8:** Regression results of SLM, SEM, and SDM.

Variables	SLM-FE			SEM-FE			SDM-FE		
	Coef.	Std. Err.	*z*	Coef.	Std. Err.	*z*	Coef.	Std. Err.	*z*
*lnGDP*	0.033	0.048	0.69	0.047	0.047	1.01	0.034	0.048	0.71
*ln*Income	0.629^***^	0.205	3.08	0.531^**^	0.224	2.37	0.412^*^	0.225	1.83
*lnPD*	−0.413^***^	0.088	−4.68	−0.482^***^	0.094	−5.12	−0.491^***^	0.096	−5.09
PG	0.002	0.031	1.05	0.001	0.002	0.4	0.001	0.003	0.5
*ln*Fiscal	0.032	0.035	1.03	0.022	0.032	0.69	0.023	0.032	0.72
*lnBed*	0.425^***^	0.002	12.11	0.398^***^	0.035	11.26	0.406^***^	0.035	11.47
W*lnGDP							−0.158	0.110	−1.43
W*lnIncome							0.766^**^	0.359	2.13
W*lnPD							0.116	0.180	0.64
W*PG							0.002	0.004	0.59
W*lnFiscal							0.102^*^	0.061	1.68
W*lnBed							0.149^*^	0.083	1.79
*ρ*	0.396^***^	0.031	12.63				0.376^***^	0.035	10.64
*λ*				0.418^***^	0.034	12.2			
*σ* ^*2*^	0.045^***^	0.002	22.47	0.045^***^	0.002	22.4	0.044^***^	0.002	22.46
*N*_obs	1030			1030			1030		
*R* ^*2*^	0.211			0.116			0.228		
*Log-L*	121.419			115.174			128.515		

***p ＜ 0.01, **p ＜ 0.05, *p ＜ 0.1.

#### Analysis of regression results from the SDM.

According to the results of the SDM presented in Table 8, the regression coefficients for *ln*Income and *ln*Bed were both positive and statistically significant. This indicates that higher income or a greater number of beds in a county can contribute to improving the allocation of health workforce. In contrast, the regression coefficient for *ln*PD was negative and significant, suggesting that higher population density may inhibit the allocation of health resources. Additionally, the coefficients for the spatial lag terms of W*lnIncome and W*lnBed were positive and significant. This finding implies that increases in income or the number of beds in neighboring areas can enhance the allocation of health resources in a county. The spatial autoregressive coefficient (*ρ*) in the SDM model was 0.376 and significant, indicating a positive spatial dependence among counties in Inner Mongolia. Specifically, improvements in the allocation of health workforce in neighboring counties had a positive impact on the given county.

To interpret the results more accurately, we followed the approach of Parent and LeSage [50] to decompose the total effects of each independent variable into direct and indirect effects. The detailed decomposition results were presented in Table 9.

**Table 9:** Spatial effect decomposition of SDM with a two-way fixed effects.

Variables	Direct effects	Indirect effects	Total effects
*lnGDP*	0.023(0.430)	−0.216(−1.200)	−0.193(−0.920)
*ln*Income	0.486^**^(2.270)	1.376^***^(2.860)	1.862^***^(3.630)
*lnPD*	−0.486^***^(−5.220)	−0.087(−0.330)	−0.574^**^(−1.990)
PG	0.002(0.650)	0.004(0.810)	0.006(1.060)
*ln*Fiscal	0.033(1.020)	0.169^*^(1.860)	0.202^**^(1.980)
*lnBed*	0.435^***^(11.920)	0.454^***^(4.020)	0.889^***^(6.880)

Socioeconomic, demographic factors, institutional environments, and supportive resources had different degrees of influence on HW. Since both the dependent variable and most independent variables were log-transformed, the coefficients can be interpreted as elasticities. Specifically, regarding direct effects, a 1% increase in disposable income per capita and bed density were associated with increases of 0.486% and 0.435% in the HW of that county, respectively. Conversely, population density showed a significant negative effect, a 1% increase in population density led to a 0.486% decrease in the HW. Regarding indirect effects, disposable income, fiscal self-sufficiency ratio, and bed density were obviously positive spatial associations on HW. A 1% increase in these variables in neighboring counties resulted in increases of 1.376%, 0.169%, and 0.454% in the local county’s HW, respectively (Table 9).

#### Endogeneity.

To implement a more rigorous endogeneity verification strategy, we adopted a two-step approach. We first used a conventional non-spatial 2SLS estimation to address the endogeneity of income, followed by a spatial GS2SLS estimation to further correct the joint endogeneity of independent variables and spatially lagged covariates.

To address potential endogeneity between regional income levels and the allocation of health workforce resources, we implemented an instrumental variable (IV) strategy using external instruments that are theoretically related to economic and infrastructure conditions but exogenous to health personnel distribution. Specifically, the regional shares of the secondary and tertiary industries and road infrastructure density were employed as instruments for disposable income per capita. These variables affect local income levels and economic activity but are unlikely to directly influence health workforce allocation except through income effects, thereby satisfying the relevance and exogeneity requirements for valid instruments.

The first-stage regression results confirmed the strong relevance of the instruments: the secondary industry share was negatively associated with income (*β* = −0.226, *p* < 0.01), while tertiary industry share and road infrastructure density were positively associated (*β* = 0.101 and 0.209, both *p* < 0.01). The first-stage *F*-statistic (104.24) far exceeded the conventional threshold of 10, alleviating concerns about weak instruments. In the second stage, after correcting for potential endogeneity, disposable income (*ln*Income) remained a significant and positive determinant of the health workforce (*β* = 0.070, *p* < 0.05). However, its magnitude was substantially smaller than in the baseline regression (*β* = 0.412), suggesting that part of the earlier income effect might have been overstated due to simultaneity bias. GDP per capita remained statistically insignificant (*β* = −0.060, *p* > 0.05), consistent with the baseline conclusion that income, rather than GDP, is the primary economic driver of workforce allocation. Bed density continued to exert a significant positive effect (*β* = 0.512, *p* < 0.01), reinforcing the importance of healthcare infrastructure availability. Diagnostic tests supported the validity of the identification strategy. The Kleibergen–Paap LM statistic (143.77, *p* < 0.001) rejected the null of under-identification, while the Hansen J test (*p* = 0.765) indicated that the instruments were exogenous. The Cragg–Donald Wald *F*-statistic (130.67) also confirmed the strong joint relevance of the instruments. These findings suggest that the positive effect of income on health workforce allocation remains robust after accounting for endogeneity, although its magnitude weakens, implying that previous estimates may have been partially biased upward. In contrast, the effects of GDP per capita and health resource density remain largely consistent with the baseline results (Table 10).

**Table 10:** Instrumental variable regression results (two-stage least squares estimation).

Variables	First-Stage (*ln*Income) Coef. (Std. Err.)	Second-Stage (*ln*HW) Coef. (Std. Err.)
Instrumental Variables		
Secondary industry share	−0.226***(0.025)	
Tertiary industry share	0.101***(0.036)	
Road infrastructure	0.209***(0.026)	
Explanatory variables		
*ln*Income		0.070**(0.045)
*lnGDP*	0.111**(0.030)	−0.060(0.040)
*lnPD*	0.060(0.062)	−0.557(0.113)
PG	−0.140*(0.021)	0.031(0.036)
*ln*Fiscal	0.003*(0.002)	0.003(0.002)
*lnBed*	0.274**(0.023)	0.512***(0.071)
Diagnostic Tests		
*N*_obs	1,030	1,030
First-stage F-statistic	104.24	–
Kleibergen–Paap LM stat	143.77 (*p* < 0.001)	–
Hansen J test (*p*-value)	–	0.765
Cragg–Donald Wald F	130.67	–

We further strengthened the endogeneity assessment by incorporating the spatial structure of the model. Specifically, to account for the potential joint endogeneity of independent variables and its spatially lagged counterpart inherent in the SDM we implemented a generalized spatial two-stage least squares estimator (GS2SLS). Using a standardized first-order spatial weight matrix, the GS2SLS results indicate a significant and positive spatial lag of the dependent variable (*ρ*= 0.395, *p* < 0.01), suggesting notable spatial dependence in health workforce allocation. The Rho F-test further confirmed the significance of spatial dependence (*F* = 11.511, *p* = 0.0007). The overall model fit is also satisfactory, as reflected by a highly significant model F-test (*F* = 51.24, *p* < 0.001). After jointly instrumenting, disposable income remained positive and statistically significant (*β* = 0.162, *p* < 0.05), while bed density and population density continued to exert strong effects. These findings show that accounting for spatial dependence and the endogeneity of spatially lagged covariates does not alter the main conclusions of the analysis. The GS2SLS estimates are presented in Table 11.

**Table 11:** Spatial IV estimation using GS2SLS.

Variables	GS2SLSXT (*ln*HW) Coef. (Std. Err.)
*W*·*ln*HW	0.395***(0.116)
*ln*Income	0.162**(0.088)
*lnGDP*	−0.034(0.041)
*lnPD*	−0.515***(0.150)
PG	0.003(0.002)
*ln*Fiscal	0.066(0.045)
*lnBed*	0.396***(0.096)
Constant	2.203(1.065)

#### Robustness checks.

To ensure the robustness of our findings, we conducted a series of sensitivity analyses using alternative model specifications. First, to test structural robustness, we replaced the original spatial weight matrix with several alternative forms, including inverse-distance, exponential-decay economic–geographic, economic-distance adjusted, and nested economic–geographic matrices. As shown in Table 12, the main results remained largely consistent across different spatial weight specifications. In particular, the direct effects of *ln*Income and *ln*Bed were robustly positive, while *ln*PD consistently exhibited a significant negative direct effect, indicating that higher disposable income and greater hospital bed availability are associated with better local health workforce allocation, whereas population density continues to constrain it. Moreover, the indirect effects of *ln*Income, *ln*Fiscal, and *ln*Bed generally remained positive and significant, reinforcing the presence of spatial dependence in health workforce distribution. We also assessed the sensitivity to the decay parameter *β*, which controls the rate at which spatial influence attenuates with distance. As reported in Table 13, the results were stable under alternative parameter values (*β*= 0.5, 2.0). Key findings, such as the strong positive effects of income and bed availability and the negative impact of population density, remained statistically significant, confirming that our conclusions are not dependent on an arbitrary parameter choice. Second, to verify temporal robustness and potential feedback effects, we re-estimated the spatial Durbin model using one-period-lagged independent variables. The lagged model passed the spatial autocorrelation test (*ρ* = 0.223, *p* < 0.001), confirming significant spatial dependence. Compared with the baseline model, the effects of disposable income, population density, fiscal self-sufficiency, and bed density remained consistent, while GDP per capita lost significance, suggesting that its baseline effect may partly reflect endogeneity bias. In terms of spatial effects, whereas the baseline model identified significant spatial dependence for income, fiscal capacity, and bed density, the lagged-variable model showed no statistically significant indirect effects (*p* > 0.05). This indicates that the spatial associations of neighboring counties’ characteristics may weaken once temporal precedence is introduced. Nonetheless, the spatial autoregressive coefficient (*ρ*) remained significantly positive in both models, confirming the stability of spatial dependence in the distribution of the health workforce (see Table 14). Finally, to check sample sensitivity, we further conducted an additional robustness test by excluding Hohhot, the provincial capital, which accounts for a disproportionate concentration of medical resources. After removing its 9 districts/counties from the sample, the results (see Table 15) remained qualitatively similar. The direct positive effects of *ln*Income and *ln*Bed, the negative impact of *ln*PD, and the positive indirects of *ln*Fiscal and *ln*Bed were still observed, indicating that the baseline results were not driven by the outlier influence of Hohhot.

**Table 12:** Robustness checks using different specifications of the spatial weight matrix.

Spatial weight matrix	Variables	Direct effects	Indirect effects	Total effects
$w_{ij}^{\left(1\right)}$	*lnGDP*	0.018 (0.310)	−0.201 (−1.110)	−0.183 (−0.850)
	*ln*Income	0.472**(2.180)	1.352***(2.780)	1.824***(3.550)
	*lnPD*	−0.493***(−5.18)	−0.095 (−0.370)	−0.588**(−2.01)
	PG	0.003 (0.720)	0.005 (0.830)	0.008 (1.130)
	*ln*Fiscal	0.029 (0.950)	0.162*(1.770)	0.191*(1.920)
	*lnBed*	0.428***(11.70)	0.447***(3.980)	0.875***(6.750)
$w_{ij}^{\left(2\right)}$	*lnGDP*	0.008(0.16)	0.02(0.33)	0.029(0.32)
	*ln*Income	0.705***(3.33)	0.690***(3.13)	1.39***(4.55)
	*lnPD*	−0.509***(−5.48)	0.174(1.81)	−0.335***(−2.48)
	PG	0.003(1.12)	−0.002(−0.77)	0.0008(0.24)
	*ln*Fiscal	0.035(1.05)	0.033(0.88)	0.067(1.25)
	*lnBed*	0.453***(12.3)	0.132***(3.34)	0.585***(9.71)
$w_{ij}^{\left(3\right)}$	*lnGDP*	0.0168(0.32)	0.342(1.81)	0.359(1.71)
	*ln*Income	0.723***(3.45)	0.833(1.44)	1.556**(2.45)
	*lnPD*	−0.537***(−5.87)	0.327(1.33)	−0.211(−0.77)
	PG	0.003(1.32)	−0.001(−0.28)	0.002 (0.29)
	*ln*Fiscal	0.025(0.77)	−0.059(−0.41)	−0.034(−0.21)
	*lnBed*	0.429***(11.66)	0.225**(1.94)	0.654***(4.89)
$w_{ij}^{\left(4\right)}$	*lnGDP*	0.024(0.46)	0.130(1.11)	0.154(1.31)
	*ln*Income	0.835***(3.99)	4.057*(1.62)	4.893**(1.93)
	*lnPD*	−0.543***(−5.95)	1.973(1.89)	1.430(1.36)
	PG	0.003(1.3)	−0.001(−0.09)	0.002(0.1)
	*ln*Fiscal	0.033(0.99)	0.158(0.25)	0.191(0.29)
	*lnBed*	0.439***(11.84)	1.195**(2.51)	1.635***(3.34)

**Table 13:** Robustness checks under alternative values of the decay parameter (*β*).

β	Variables	Direct effects	Indirect effects	Total effects
β=2	*lnGDP*	0.030(0.58)	−0.064(−0.51)	−0.034(−0.22)
	*ln*Income	0.507***(2.41)	0.989***(2.71)	1.497***(3.56)
	*lnPD*	−0.498***(−5.44)	0.069(0.36)	−0.430**(−1.95)
	PG	0.002(0.8)	0.001(0.37)	0.003(0.77)
	*ln*Fiscal	0.035(1.09)	0.098(1.43)	0.133*(1.64)
	*lnBed*	0.434***(11.84)	0.297***(3.59)	0.731***(7.2)
β=0.5	*lnGDP*	0.019(0.33)	−0.334(−1.38)	−0.316(−1.16)
	*ln*Income	0.646***(2.92)	1.186**(1.9)	1.832***(2.9)
	*lnPD*	−0.439***(−4.61)	−0.195(−0.59)	−0.633**(−1.77)
	PG	0.001(0.5)	0.006(1)	0.007(1.18)
	*ln*Fiscal	0.033(1.01)	0.304**(2.55)	0.300**(2.51)
	*lnBed*	0.454***(12.29)	0.608***(4.17)	1.063***(6.66)

**Table 14:** Estimation results of SDM with lagged independent variables.

Variables	Direct effects	Indirect effects	Total effects
*lnGDP*	0.002(0.06)	0.104(1.52)	0.107(1.55)
*ln*Income	0.444**(2.43)	0.181(0.94)	0.624***(8.27)
*lnPD*	−0.175**(2.13)	−0.062(−0.36)	−0.113(−0.68)
PG	0.0009(0.46)	0.0004(0.13)	0.001(0.4)
*ln*Fiscal	0.069**(2.55)	0.079(1.49)	0.148***(2.76)
*lnBed*	0.091***(2.93)	0.091(1.16)	0.183**(2.07)

**Table 15:** Robustness checks of model estimates after excluding Hohhot (9 districts/counties).

Variables	Direct effects	Indirect effects	Total effects
*lnGDP*	−0.018(−0.33)	−0.186(−1.04)	−0.204(−0.97)
*ln*Income	0.420**(1.95)	1.645***(3.44)	2.065***(4)
*lnPD*	−0.685***(−7.24)	−0.025(−0.09)	−0.710**(−2.34)
PG	0.002(0.99)	0.005(0.89)	0.007 (1.22)
*ln*Fiscal	0.037(1.14)	0.194**(2.14)	0.231**(2.24)
*lnBed*	0.367***(9.64)	0.530**(4.34)	0.897***(6.42)

#### Heterogeneity analysis.

To further examine whether the determinants of the health workforce distribution differ between municipal and non-municipal counties, interaction terms between key independent variables and the dummy variable CityDistrict (1 = municipal county, 0 = non-municipal county) were introduced into the SDM model. The estimation results of the two-way fixed effects SDM with interaction terms were presented in Table 16.

**Table 16:** Direct, indirect, and total effects of key determinants on health workforce allocation with interaction terms (CityDistrict = 0 as reference).

Variables	Direct effects	Indirect effects	Total effects
*lnGDP*	0.0004(0.01)	−0.199(−1.24)	−0.199(−1.05)
*ln*Income	0.438***(4.68)	0.848***(3.19)	1.286***(4.46)
*lnPD*	−1.328***(−4.73)	1.281**(1.88)	−0.046(−0.06)
PG	0.002(0.79)	−0.001(−0.22)	0.0008(0.15)
*ln*Fiscal	−0.005(−0.15)	0.092(1.14)	0.088(0.96)
*lnBed*	0.177***(6.29)	0.542***(6.14)	0.719***(7.44)
CityDistrict×*ln*Income	0.147***(3.6)	0.556***(4.39)	0.703***(4.98)
CityDistrict×*lnPD*	0.670**(2.08)	−1.834**(−2.16)	−1.164(−1.31)
CityDistrict×*ln*Fiscal	0.133**(2.2)	0.138(0.63)	0.271(1.12)
CityDistrict×*lnBed*	0.162***(4.1)	−0.584***(−4.08)	−0.421***(−2.65)

The results showed that the model remains robust and stable, with a significantly positive spatial autoregressive coefficient (*ρ* = 0.365, *p* < 0.01), indicating a moderate spatial dependence in the distribution of the health workforce. Moreover, the estimated directions and significance levels of the main independent variables were consistent with those in the baseline model, further confirming the robustness of the findings. Notably, the interaction terms revealed clear spatial heterogeneity between municipal and non-municipal counties (CityDistrict = 0 as reference). The coefficients of CityDistrict×*ln*Income and CityDistrict×*lnBed* were positive and statistically significant, suggesting that the positive effects of income level and hospital bed density on the health workforce are more pronounced in municipal counties. In contrast, CityDistrict×*lnPD* showed a significantly positive direct effect but a negative indirect effect, implying that higher population density enhances local workforce aggregation while weakening spatial dependence in municipal areas. Although the direct effect of CityDistrict×*ln*Fiscal was significant, its indirect and total effects were insignificant, indicating that fiscal self-sufficiency exerts a broadly similar influence across different county types. These findings confirm the existence of spatial heterogeneity in the determinants of health workforce allocation. Municipal counties are more effective in attracting and retaining health professionals, while non-municipal counties face greater constraints.

## Discussion

From 2013 to 2022, the number of health professionals, physicians, and nurses per 1,000 population in Inner Mongolia exhibited an increasing trend, with the most notable growth observed among nurses, particularly after 2016. This trend reversed the previously inverted physician-to-nurse ratio. The change can primarily be attributed to policy support and economic development in Inner Mongolia. The “13th Five-Year Plan for Deepening the Reform of the Medical and Health System”(2016) emphasized strengthening primary healthcare services and improving nursing education and training, which significantly expanded the nursing workforce. [52]. These measures include increased funding for nursing education, expanded in-service training, and enhanced collaboration with medical institutions to improve the professional skills and service capabilities [22]. The “Healthy China 2030” blueprint (2016), further emphasized the importance of nurses by promoting professional development and introducing compensation systems tailored to the healthcare sector. These strategies were incorporated into the “14th Five-Year Plan for Inner Mongolia” (2021), which prioritized expanding formal staffing and improving the working conditions, especially nurses. Second, the rising economy and healthcare needs, particularly in response to the increasing demand for chronic disease management and elderly care due to an aging population, have further highlighted the importance of nurses, thereby driving the demand for nurses [21]. Despite improvements in the physician-to-nurse ratio, the results remain below the national average [21,52,53]. This phenomenon is linked to factors such as limited formal staffing quotas, high workload and stress levels, relatively low salary and benefits, and insufficient career development opportunities [22,33]. To achieve a more balanced physician-to-nurse ratio and enhance the effectiveness of nursing services, hospitals should adopt comprehensive measures, including increasing nurses’ salaries and benefits to reduce turnover, expanding formal staffing quotas to lower attrition risks, alleviating workload pressures, eliminating salary disparities between formally and contractually employed nurses to enhance fairness and job satisfaction [20–22]. Additionally, effective incentive mechanisms should be established through strengthening performance-oriented management and improving the promotion system [21]. Meanwhile, the government should provide policy support to nurses and work to change the traditional emphasis on medical treatment over nursing in healthcare institutions, emphasizing the role and value of nurses in medical services [52].

We found that there was a significant difference in the number of health professionals per 1,000 population at the county level in Inner Mongolia in 2013–2022. Areas with higher densities were partly located in the central regions of economically developed leagues and cities, such as Hohhot and Baotou. Due to high incomes and abundant career development opportunities, they have a great attraction for health workforce [18,19]. Another part was located in sparsely populated regions, such as Alxa League in the west and Hulunbuir City in the northeast. In these areas, the relatively small population size results in a high density of health professionals [19,20]. Over time, the differences in health workforce among regions have shown a decreasing trend, consistent with previous research findings [20,53]. This is mainly attributed to the support of a series of government policies. For example, “Opinions of the Central Committee of the Communist Party of China and the State Council on Deepening the Reform of the Medical and Health System”, “The 14th Five - Year Plan for the National Economic and Social Development of Inner Mongolia and the Long - Term Goals for 2035”, and “The Implementation Plan for ‘Healthy Inner Mongolia 2030’” have jointly promoted balanced development, increased financial support for remote and underdeveloped areas, and ensured that these areas obtain sufficient medical resources [10,52]. At the same time, the government has implemented standardized training for resident physicians and free medical education initiatives that require graduates to work in designated rural and pastoral areas, attracting more healthcare professionals to resource scarce regions and enhancing local healthcare service capacity [5,10,24,37,54]. In addition, the promotion of telemedicine services and the advancement of public health projects have improved primary healthcare services, further reducing regional disparities [37,53].

We used spatial autocorrelation analysis combined with an economic-geographic nested weight matrix to reveal the spatial patterns of health workforce distribution at the county level in Inner Mongolia from 2013 to 2022. Through global spatial autocorrelation analysis and Moran’s scatterplots, it was found that the positive spatial clustering of health workers gradually weakened, and the spatial distribution of health workforce gradually changed from an initially obvious clustering to a more balanced state. This further verifies that the differences in health workforce among regions are gradually narrowing.

Local spatial correlation analysis revealed the hotspots and coldspots of the health workforce density in Inner Mongolia. The hotspots were concentrated in the economic centers such as Yuquan District, Huimin District, and Saihan District in Hohhot, Dongsheng District and Kangbashi District in Ordos, and Qingshan District and Jiuyuan District in Baotou. These areas have high GDP and per capita income. Their strong economic strength provides a solid financial foundation for the development of healthcare, supporting the construction of more medical institutions and the investment in health workforce [19,20]. Additionally, economic prosperity has created high-quality job opportunities and career advancement prospects, attracting a large number of health professionals to gather [7]. The dense population in these regions also drives higher demand for medical services, necessitating sufficient allocation of health workforce [45]. In contrast, the coldspots were mainly located in the rural or pastoral areas in central Inner Mongolia, such as Taibus Banner in Xilingol League, Zhuozi County and Chahar Right Back Banner in Ulanqab City. These areas have relatively low economic levels, lacking sufficient funding for the development of healthcare infrastructure, which results in a shortage of health resources. The limited career development opportunities make it difficult to attract and retain high-quality health professionals [40]. Moreover, many coldspots were geographically remote with poor transportation, increasing the challenges of resource allocation and reducing the attractiveness to health professionals. To promote equitable distribution of the health workforce, targeted interventions are needed in underserved rural and pastoral regions. Drawing on Australia’s rural general practitioner (GP) policy, which offers financial incentives, rural training pathways, and long-term retention strategies to attract and retain medical personnel [55]. It is recommended that the government increase financial investment in underdeveloped rural and pastoral areas, particularly the coldspots [4,20]. Special funds should be established to prioritize the construction and improvement of healthcare infrastructure, ensuring the availability of essential medical equipment. Policies should be formulated to attract professionals, such as providing housing subsidies, preferential treatment for professional title promotion, and children’s education support. Collaborations with universities could facilitate targeted training programs for health professionals, establishing long-term mechanisms for continuous professional development through regular advanced training for grassroots health workers [22,40]. Furthermore, strengthening the informatization of healthcare services can help share high-quality resources via telemedicine [37,40,53]. Regional cooperation mechanisms for healthcare, such as medical alliances, shared telemedicine, and joint workforce training, should also be established to enhance collaboration and resource sharing among neighboring area and support a more equitable distribution of the health workforce [19,20].

This study further employed a spatial panel econometric model to identify the key factors influencing the distribution differences of health workforce and their spatial dependence. The results showed that disposable income per capita, bed density, and population density were critical factors affecting the allocation of HW. Among them, disposable income per capita was positively correlated with HW, and this view has been widely confirmed in previous studies [20,36]. In regions with better economic conditions and higher income, medical institutions can offer more competitive salaries, which not only helps attract health professionals but also effectively reduces workers loss, particularly in rural and remote areas. This factor is also a key driver of healthcare worker mobility both domestically and internationally [24,56]. According to Benchimol and Qureshi [46] economic preferences evolve with changes in income and development levels, shaping how societies allocate resources over time. As disposable income increases, both individuals and governments tend to shift their priorities from basic subsistence needs toward higher-quality and specialized health services. This dynamic adjustment in economic preferences provides an underlying mechanism linking regional income growth to improved attraction and retention of the health workforce and further explains why income growth is closely associated with workforce concentration in more developed areas. Bed density also exerted a positive impact on HW, consistent with prior findings [33]. The number of beds reflects the availability of inpatient services and the service capacity of medical institutions [56]. Higher bed density can meet the treatment needs of more patients while requiring an increase in the number of health workers to ensure service quality [33]. Moreover, such regions often possess advanced medical equipment, well-developed facilities, and substantial funding for talent recruitment, all of which contribute to the growth of health professionals [57]. Notably, population density exhibited a negative impact on HW. From an economic perspective, market mechanisms suggest that higher population density leads to greater demand for health services, which should result in a larger allocation of health workers. However, in reality, under similar distributions of medical institutions, higher population density increases the accessibility of healthcare services for residents, meaning fewer medical institutions may be required to serve the same population. In contrast, sparsely populated areas with the same population require more medical institutions and health workers to ensure the accessibility of healthcare services [57,58].

Interestingly, the results of the indirect effects in the SDM indicated that disposable income per capita, bed density, and fiscal self-sufficiency rate all had positive spatial associations on HW. This can be attributed to three main reasons: Firstly, economic interdependence plays a significant role. When disposable income per capita and fiscal self-sufficiency rates increase, the regional economic strength is enhanced [35]. This not only improves local health resource allocation but also promotes regional economic interaction, driving surrounding areas to invest more in health workforce through economic collaboration [33,57]. Second, demand-driven healthcare services contribute to this effect. An increase in bed density enhances the capacity of medical services, attracting more patients, including those from neighboring regions [58]. This creates healthy competition, encouraging surrounding areas to increase their health workforce to meet growing service demands. Third, resource radiation and cooperation foster mutual benefits. The financial investment brought about by improved economic strength, along with advanced medical equipment and technologies in high-bed-density regions, can be shared with neighboring areas through technical exchanges and medical training [36]. This forms a win-win, promoting both the quality and quantity of health workforce in surrounding regions. During 2013–2022, with the economic development, disposable income per capita in Inner Mongolia increased steadily, and fiscal self-sufficiency rates in some areas remained at a relatively high level. The core economic zone of Hohhot – Baotou – Ordos leveraged industrial cooperation and professionals mobility to boost the economic growth of neighboring regions such as Ulanqab and Bayannur. Alongside economic progress, these regions increased their investment in healthcare, constructing and expanding hospitals, thereby raising bed density. The improvement in healthcare service capabilities in cities like Hohhot and Erdos attracted patients from surrounding areas, prompting these regions to correspondingly increase their health workforce to meet rising demand. For example, in the Hohhot – Baotou – Ordos region, HW increased from 6.39 in 2013 to 9.27 in 2022. Similarly, in Ulanqab, this figure rose from 4.04 in 2013 to 7.87 in 2022, with a growth rate of 95.00% [6]. Moreover, advanced medical equipment and technologies are disseminated to surrounding regions through regional collaborations and telemedicine services. Especially after 2018 and 2019, when Inner Mongolia began constructing urban medical treatment partnership system and an integrated county-level medical communities, characterized by vertical linkage, complementary advantages, and shared construction, the technical level of healthcare in surrounding areas was significantly improved [59]. This further promoted the overall quality and quantity of health workforce across the region.

In addition, the extended analyses addressing endogeneity, robustness, and spatial heterogeneity further reinforce the reliability of our findings. After controlling for potential simultaneity between income and workforce allocation through the instrumental variable approach, the positive effect of disposable income remained significant though attenuated, suggesting that baseline estimates may have slightly overstated its magnitude due to endogeneity. Robustness tests using alternative spatial weight matrices, varying decay parameters, lagged independent variables, and the exclusion of Hohhot consistently confirmed the stability of the main relationships—particularly the positive impacts of income and bed density and the negative effect of population density—indicating that the results are not sensitive to model specification, spatial structure, or sample composition. Furthermore, the heterogeneity analysis based on municipal and non-municipal subsamples revealed significant spatial variation: economic capacity and healthcare infrastructure exert stronger effects in municipal counties, while non-municipal counties face greater difficulty in attracting and retaining health workers. These heterogeneous effects align with labor market theory, spatial equilibrium theory, and established frameworks of health workforce allocation. Municipal counties, with stronger fiscal capacity and better healthcare infrastructure, offer more competitive compensation, career opportunities, and living conditions, thereby attracting and retaining more health professionals [7,13]. From a spatial equilibrium perspective, these advantages lower transaction and mobility costs, reinforcing workforce concentration in developed areas. In contrast, non-municipal counties face structural constraints such as weaker fiscal autonomy and limited healthcare facilities, which act as “push factors” that hinder workforce inflow despite policy incentives. As noted by Benchimol and Qureshi [46] and Asamani, et al. [41], insufficient economic and fiscal capacity restricts investment in human resource development, sustaining regional disparities in workforce distribution. Collectively, these additional tests demonstrate that the observed spatial dependence and key determinants of health workforce allocation are structurally robust, lending greater credibility to the empirical conclusions.

To optimize the allocation of health workforce in Inner Mongolia from the perspective of the spatial econometric model, efforts should be made in multiple aspects. Firstly, in terms of economic development and policy support, efforts should focus on promoting regional economic synergy. Measures such as establishing regional industrial cooperation funds, providing fiscal subsidies and tax incentives, and encouraging joint investment can enhance the economic interactions from core areas (e.g., Hohhot-Baotou-Ordos) to surrounding regions. This can enhance disposable income per capita in these regions and lay a solid economic foundation for the development of health workforce [19,20]. However, if such economic policies are not carefully designed, they may inadvertently exacerbate urban–rural disparities. Therefore, targeted strategies must ensure that growth dividends benefit underserved regions, avoiding the unintended consequence of deepening inequalities. Additionally, addressing income disparities among health workers between urban and rural areas, as well as across regions, is critical [17,56]. By aligning income with professional value, the attractiveness of positions in underdeveloped areas can be improved, encouraging more professionals to work in these regions [56]. Secondly, regarding resource optimization, health workforce planning should take into account population size, geographic conditions, and economic status, while integrating regional health service demands and the current distribution of health professionals [17–19]. Using big data technology to conduct precise analyses of factors such as aging and population flow can facilitate dynamic adjustments in resource allocation. Furthermore, Geographic Information Systems (GIS) can be introduced to effectively utilize spatial data mapping for the planning, allocation, and monitoring of health resources thereby ensuring that distribution is aligned with demand, particularly in low-resource regions [26]. Thirdly, in enhancing regional cooperation and resource sharing, the construction of urban medical treatment partnership system and an integrated county-level medical communities should be advanced [18,19]. These initiatives aim to improve the sharing of medical resources, promote the inflow of high-quality healthcare resources to grassroots levels, and reduce urban-rural disparities. Internal health professionals mobility, technical exchanges, and resource sharing within healthcare alliances and networks should also be strengthened [35,40,53]. Measures such as telemedicine, expert consultations, and technical training can enhance the service capabilities of grassroots medical institutions and improve the quality of health human resources at the local level [40,52,53]. Lastly, improving fiscal investment should be complemented by the enhancement of institutional expertise. According to Asamani, et al. [41], unless health workforce is given sufficient priority in government health expenditure (at least reaching the global average level of 57%), the persistent shortage of health workers resulting from long-term underinvestment will remain difficult to resolve. As noted by Benchimol, et al. [43] and Benchimol and Qureshi [46], institutional expertise—the capacity to design, manage, and carry out effective health policies—plays a decisive role in translating financial inputs into improved health workforce outcomes. Regions with similar fiscal capacity may therefore experience very different results depending on the strength of their administrative and governance capabilities. Our results align with this perspective to some extent: while income and fiscal resources promote health workforce growth, persistent disparities suggest that institutional capacity may also play a role in shaping how effectively these resources are translated into workforce improvements. Therefore, in addition to increasing financial support and transfer payments to primary healthcare institutions in remote areas, governments should also focus on building local administrative capacity. This includes strengthening local health departments in planning, monitoring, and implementation capabilities to ensure that financial resources lead to sustainable improvements in infrastructure, personnel training, and service delivery [20,52]. In addition, evaluating the potential returns on investments in remote areas is essential for effective policy design. This includes not only assessing short-term service improvements but also considering the long-term sustainability and equity of investments. Policymakers should also be mindful of possible trade-offs, such as the diversion of resources from urban centers or the over-reliance on subsidies. Strategic planning that balances efficiency and equity is necessary to avoid reinforcing structural disparities while improving workforce allocation across the region.

Our study has several limitations. First, this research utilized the commonly adopted international indicator of health workforce—the number of health professionals per 1,000 population—to measure the allocation of health workforce in Inner Mongolia. While this indicator primarily reflects fairness in population distribution, it partially overlooks fairness in geographic distribution [37,53]. Second, due to data limitations, several potentially important factors such as age structure, urbanization, and education expenditure were not included. Individual characteristics like age and education may also affect health worker allocation but were beyond the scope of this study. Third, due to data constraints, we did not apply causal inference methods, and therefore the reported associations should not be interpreted as causal effects. Future research should address these methodological concerns and incorporate additional variables to deepen our understanding of the mechanisms influencing health workforce distribution.

## Conclusion

This study examines the spatial distribution and influencing factors of the health workforce (HW) in Inner Mongolia from 2013 to 2022, using spatial correlation analysis and a spatial Durbin model. Our findings reveal that although HW distribution disparities persist, spatial clustering has weakened over time. Disposable income, bed density, and fiscal self-sufficiency positively affect HW allocation and exhibit notable spatial associations, while population density has a negative impact. These findings suggest that improving regional economic synergy, investing in underserved rural and pastoral areas, and enhancing local governance are essential to promoting equitable HW allocation. Strengthening medical alliances and integrated service networks can also improve resource sharing across regions. This study contributes by highlighting both local and neighboring influences on HW distribution. Future research should explore geographic accessibility and include individual-level factors to deepen understanding of HW allocation patterns.

## Supporting information

S1 Table Variable definitions, transformations, and data sources. (PDF)

S1 Fig Spatial distribution of independent variables in Inner Mongolia, 2013–2022. (TIF)

S2 Fig Temporal trends of key independent variables in Inner Mongolia, 2013–2022. (TIF)
