# Supplementary material for: Spatial econometric analysis of health workforce distribution and its influencing factors in Inner Mongolia, China
Source: PLoS One. 2026 Jan 20;21(1):e0340381. doi: 10.1371/journal.pone.0340381 (PMC12818611; doi:10.1371/journal.pone.0340381)
Supplement: S1 Table — (PDF) [file pone.0340381.s001.pdf]

S1 Table Variable definitions, transformations, and data sources

Variable	Definition	Transformation	Unit	Source
Health workforce (HW)	Number of health professionals (physicians, nurses, and health technicians) per 1,000 population = (Total number of health professionals / total population) × 1,000	*ln*(HW)	persons/1,000	Inner Mongolia Statistical Yearbook (2013–2022)
GDP per capita (GDP)	Gross Domestic Product / total population	*ln*(GDP)	CNY/person	Inner Mongolia Statistical Yearbook (2013–2022)
Disposable income per capita (Income)	Total disposable income / total population	*ln*(Income)	CNY/person	Inner Mongolia Statistical Yearbook (2013–2022)
Population density (PD)	Total population / total land area	*ln*(PD)	persons/km²	Inner Mongolia Statistical Yearbook (2013–2022)
Population growth (PG)	(Population in year t − Population in year t−1) / (population in year t−1) × 10,000	Not transformed	per 10,000 population	Inner Mongolia Statistical Yearbook (2013 – 2022)
Fiscal self-sufficiency ratio (Fiscal)	General public budget revenue / general public budget expenditure	*ln*(Fiscal)	ratio	Inner Mongolia Statistical Yearbook (2013–2022)
Hospital beds per 1,000 population (Bed)	Total number of hospital beds / total population × 1,000	*ln*(Bed)	beds/1,000	Inner Mongolia Statistical Yearbook (2013–2022)
