# Supplementary figures and images for: Spatial econometric analysis of health workforce distribution and its influencing factors in Inner Mongolia, China
Source: PLoS One. 2026 Jan 20;21(1):e0340381. doi: 10.1371/journal.pone.0340381 (PMC12818611; doi:10.1371/journal.pone.0340381)

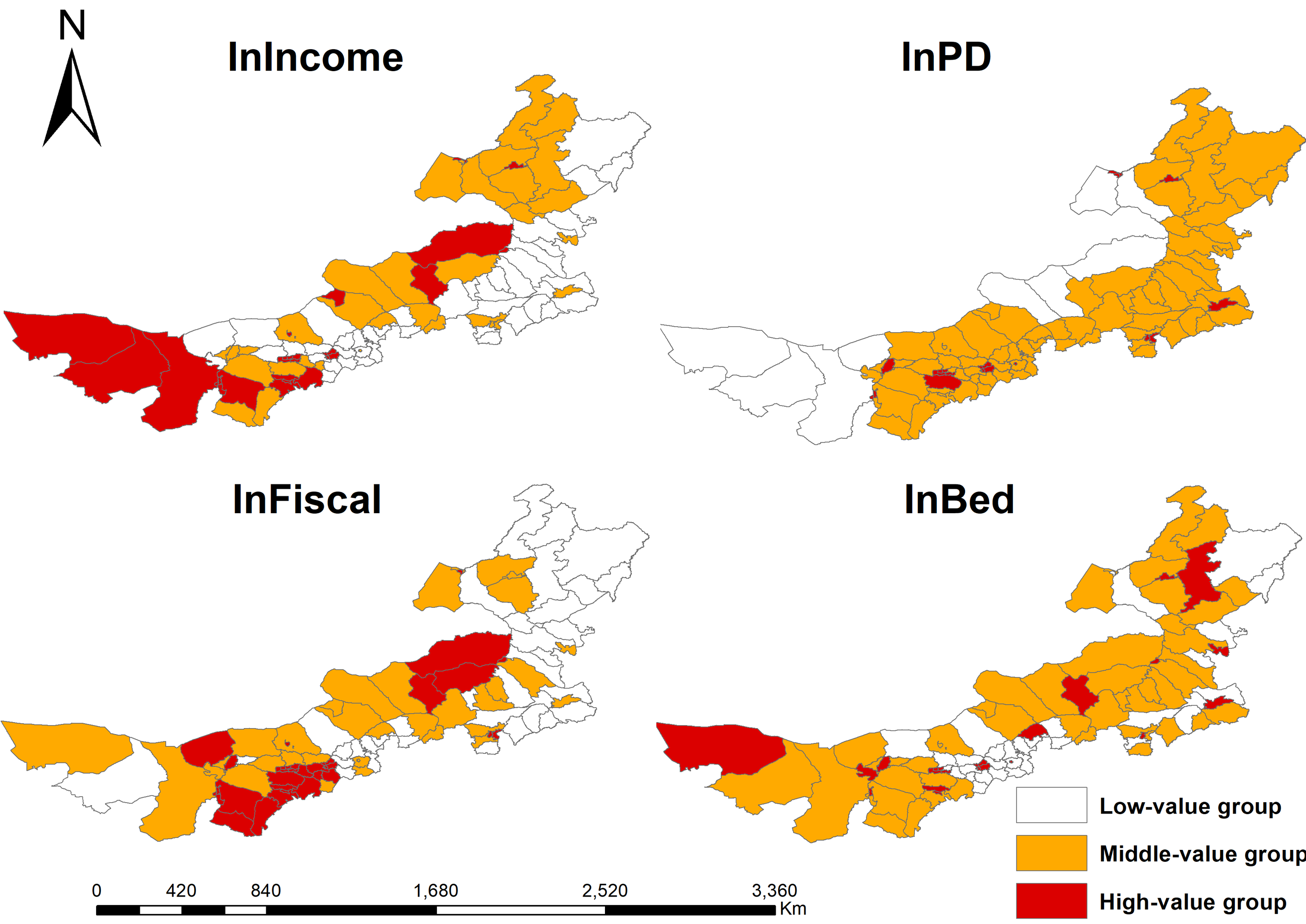

Supplement: S1 Fig — (TIF) [file pone.0340381.s002.tif]

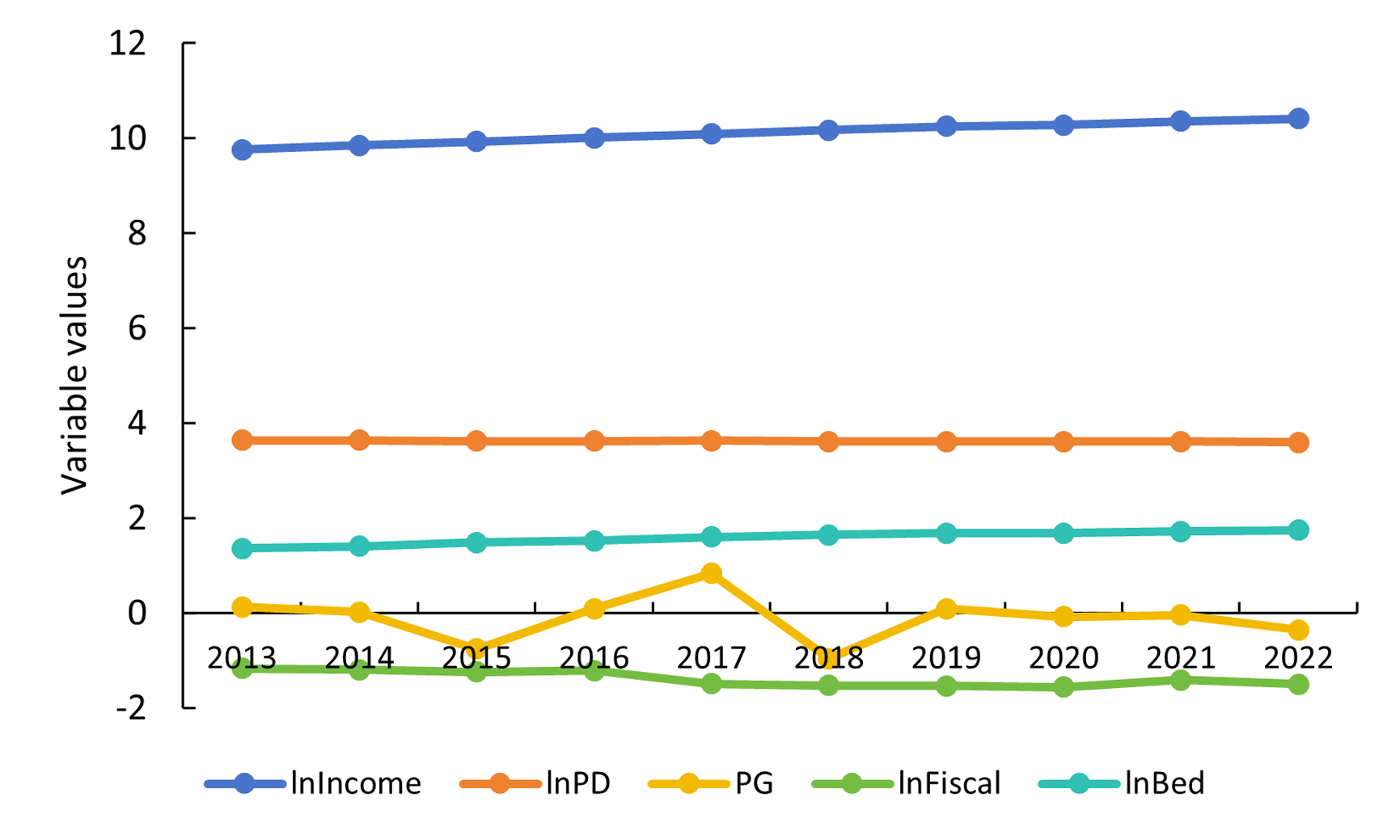

Supplement: S2 Fig — (TIF) [file pone.0340381.s003.tif]
